# Endovascular Treatment of a Right-Sided Ureteroiliac Fistula in a Patient with a Simultaneous Left-Sided Ureteroileal Fistula

**DOI:** 10.1155/2011/284505

**Published:** 2011-07-03

**Authors:** G. M. Veenstra, L. M. C. L. Fossion, G. Debonnaire, K. de Laet

**Affiliations:** ^1^Faculty of Health, Medicine and Life Sciences, Maastricht University, 6200 MD Maastricht, The Netherlands; ^2^Department of Urology, Maxima Medical Centre, De Run 4600 5504 DB, Veldhoven, The Netherlands; ^3^Department of Vascular Surgery, Maxima Medical Centre, Veldhoven, The Netherlands

## Abstract

We describe an 80-year-old female with a left ureteroileal fistula and simultaneously a right ureteroiliac fistula. Her history highlights the predisposing factors of radiation, major surgery in the region, and presence of bilateral double-J-stents. She was successfully treated with an endovascular approach after being initially misdiagnosed. There seems to be an increase in reporting ureteral fistulas, however this entity remains a rare clinical condition that can lead to life-threatening situations. A fast and accurate diagnosis of an ureteroarterial fistula remains a challenge.

## 1. Introduction

There seems to be an increased incidence of ureteroarterial fistulae (UAF), which are presumably related to improved cancer survival and aggressive multimodality treatment for abdominopelvic cancers [1]. Chronic ureteral stenting, pelvic (external beam) radiotherapy, pelvic surgery, and peripheral vascular disease are known risk factors for developing UAF [1]. A fistula between the iliac artery and ureter is considered rare; it can cause severe hematuria with need for blood transfusions and is considered a life-threatening condition. In the past UAF have mostly been treated by either surgery or a combination of surgery and arterial embolisation. Particularly in the last 10 years endovascular stenting is increasingly used instead of open techniques due to the high perioperative risk and comorbidities in patients with ureteroarterial fistulas. We present a case of such an endovascular stenting for treatment of a UAF.

## 2. Case Report

An 80-year-old female underwent a Hartmann's procedure with intraoperative radiation therapy in 2008. A metastasectomy for liver metastasis was done before the Hartmann's procedure in a separate operation. Neoadjuvant therapy included radiation therapy with a dose of 5 × 5 Gy and chemotherapy with 6 cycles of capecitabine, oxaliplatin, and bevacizumab. Since December 2009 JJ-stents were placed for ureteral stricture and functional obstruction with regularly bilateral replacement afterwards. In November 2010, extensive abscess drainage from the Douglas Cavity was needed after occluded ureteral stents with Candida glabrata infection. Furthermore she was diagnosed with Type 2 (noninsulin-dependent) diabetes mellitus and angina pectoris.

**Figure 1:**
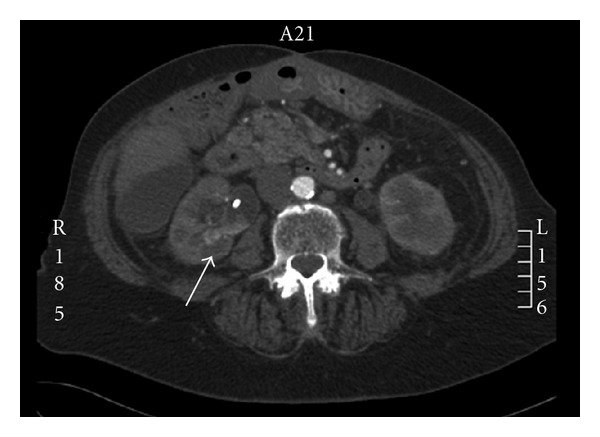
Computed tomography angiography (CTA) axial image; suspected active hemorrhage with clots in the right pyelocaliceal system.

In January 2011, she presented at the emergency room with intermittent macroscopic hematuria. An irrigation catheter was placed and manual irrigation was continued to remove all clots out of the bladder. Laboratory findings at admission included a hemoglobin level of 7,2 mmol/L and a creatinine level of 370 *μ*mol/L. Ultrasound of the kidney showed bilateral hydronephrosis. One day after admission a cystoscopy and ureteral stent exchange was performed in the operation room because of persistent hematuria and suspected occlusion of the ureteral stents. Removal of the right double-J catheter revealed a pulsatile arterial bleeding from the right ureteral orifice. The ureteral stent was quickly changed and the bleeding subsided. An emergency peroperative hemoglobin was 3,4 mmol/L (norm >7,5 mmol/L). The patient was stabilized and 4 Packed Cells were given at the OR. Open surgery was primarily not considered an option because of the patients extensive abdominal surgical history. At the radiologist intervention department, computed tomography angiography (CTA) was performed for further diagnosis (see Figure 1). 

**Figure 2:**
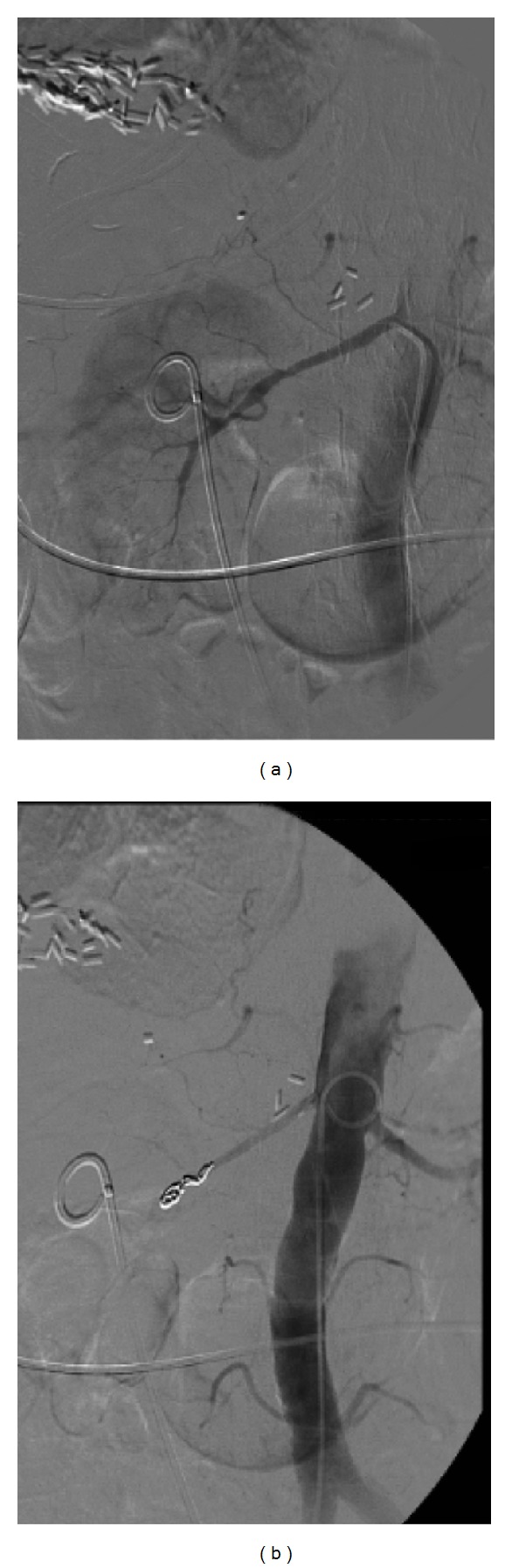
Arteriography; (a) shows the right arteria renalis. Notice the double J stent and numerous clips after the liver metastasectomy. (b) shows the coils after the embolisation of the right arteria renalis.

An active hemorrhage was suspected at the level of the right pyelum and clots were seen in the pyelocaliceal system. Since the patient was hemodynamically stable, it was decided to wait and an expectant policy was agreed. However, in the following night, the patient had gross hematuria again and embolisation of the right kidney was performed with two coils in the right renal artery (see Figure 2).

**Figure 3:**
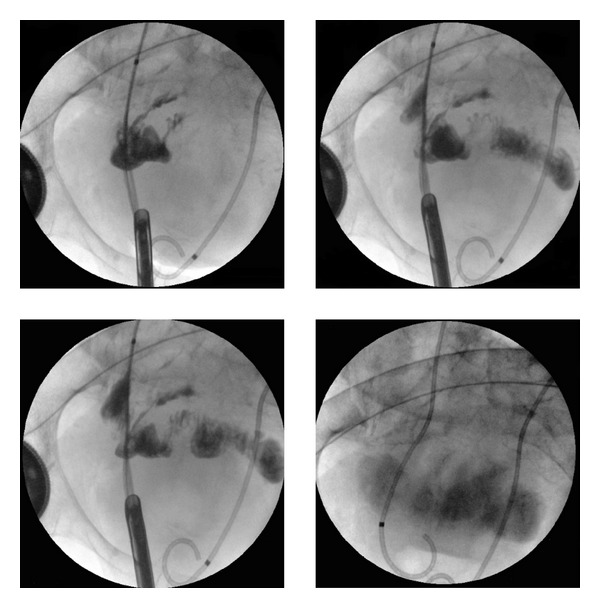
Retrograde ureterography left showing leakage of contrast in the intestines.

After embolisation, the left ureteral catheter still had to be repositioned in the operating room. A retrograde ureterography showed a ureteroileal fistula (see Figure 3). A new ureteral catheter was placed and a abdominal surgeon was consulted regarding the fistula. A conservative approach was advised. Within 24 hour massive hematuria recurred. Hemoglobin level dropped despite Packed Cells transfusion. After questioning our primary diagnosis of bleeding of the right pyelum, a fistula between the (common) iliac artery and the ureter was suspected. An ureterogram failed to visualize the fistula, so did a new CT angiography. However, on clinical grounds ureteral arterial fistula was now highly suspected. An endovascular approach was chosen since open surgical repair would be very difficult in this patient.

**Figure 4:**
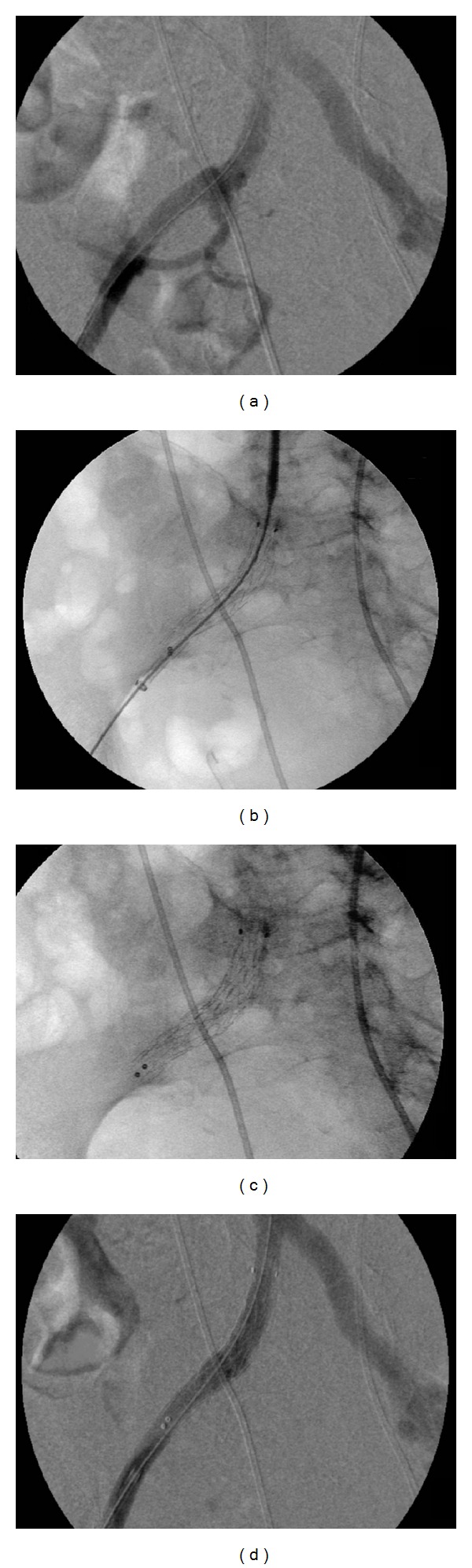
Angiography; (a) prestent placement, no extravasation of contrast into the ureter is seen. (b) endovascular stent placement over a guiding wire. (c) endovascular stent placement after removing of the guide wire. (d) Control after stent placement; no endoleakage.

Before the stent was placed an angiography of the right iliac artery failed to locate the exact position of the UAF. Under general anesthesia, a stent graft (Endurant stent type ENEW1010C80EE) was inserted via the right femoral artery and deployed at the iliac-ureteral conduct. A ureteral stent was in place to help positioning the stent graft. After placement postdilatation was done using a Reliant moulding-balloon. Intraoperative arteriography revealed no endoleakage (see Figure 4). Also in the late phase, no leakage of contrast was seen. Hematuria decreased almost instantly after the endovascular stent was placed.

After endovascular stent placement life-long antiplatelet therapy with acetylsalicylic acid was started. Antibiotic treatment was given for a period of 9 days. No prophylactic antibiotic therapy was given. Three months after the endovascular stent procedure a nephrostomy tube was placed in the left kidney because of recurrent ureteral stent occlusion. Four months after embolisation and stent placement the patient is alive with a creatinine blood level of 180 *μ*mol/L.

## 3. Discussion

Ureteroarterial fistulas usually present with gross hematuria along with flank pain in about half the cases due to obstruction of the ureter. Bleeding can be massive and dramatic, but can also be intermittent. Replacement of ureteral stents can be a precipitating event provoking hematuria [1–12]. The exact mechanism of the development of UAF is still unclear. Pressure necrosis of the catheterized ureter is believed to contribute to the formation of a fistula [3]. The pulsations of the iliac artery transmitted through an already compromised ureter to a stiff intraluminal catheter can readily produce necrosis. Previous radiation therapy and pelvic or vascular surgical procedures, may induce weakening of the ureteral and the arterial wall. When ureteral stenting is necessary for a longer period, it is therefore advisable to use small and soft silicone stents [1, 2]. 

The diagnosis of ureteroarterial fistulae can be difficult. In this case we primarily suspected the hematuria to originate from the kidney. A review from van den Bergh et al. found similar cases where an incorrect diagnosis resulted in a nephrectomy in 11 cases and embolisation of a renal artery in 4 cases [3]. Retrograde ureterography will not be of much help in a patient whose fistulous tract is temporarily closed by clots in the ureter or in the fistulous opening. Literature reports a sensitivity of a retrograde pyelogram of 45–60% [4]. Arteriography may demonstrate the site of the fistula only when it is performed during an episode of active bleeding. Overall it has a sensitivity of 23 to 41% [1, 4]. Provocative angiography, a technique to demonstrate ureteroarterial fistulae by deliberately moving the patient's indwelling stents to “unclot” the fistula while simultaneously performing angiography was not performed in our patient. In some cases it might drastically improve sensitivity [1]. However, it is not advised in unstable patients. In our patient the diagnosis was made after excluding other diagnoses because hemorrhage recurred after embolisation of the kidney and no others abnormalities were found on the CT scan or during cystoscopy. 

Endovascular stent graft repair is commonly performed for UAF. However not all reports about endovascular stenting are successful. Ando et al. described a case in Japanese with bilateral UAF where left-sided endovascular treatment failed and thromboembolism occurred in the covered stent on the right side. [5]. Occlusion of the lumen of the endovascular stent graft eight months after treatment for an UAF has been described earlier by Rodriguez et al. [6]. Krambeck et al. described marginal results with endovascular stenting in two of three patients who required secondary treatment owing to graft occlusion and continued hemorrhage [1]. Though several case reports confirmed successful use of endovascular stenting of UAF by stent grafts and demonstrated successful short-term results despite the theoretical risk of infection, occlusion, and stent-fracture after deployment of stent grafts [1, 3, 7–12]. It was therefore desirable that the long-term results of endovascular treatment by stent-grafts for uretero-arterial fistulas are reviewed [7]. Fox et al. compared endovascular treatment with open surgery with a median followup of 15.5 months (range 1 to 99). They did not identify a clear advantage for endovascular or open vascular surgical management and concluded that endovascular stenting is preferred in most cases. Particularly in inoperative patients and in acute emergency conditions an endovascular approach seems ideal [8]. If open surgical repair should follow in patients after a primary stent placement who are suitable for operation is still unclear.

## 4. Conclusion

An ureteroarterial fistula is still a rare and dangerous condition. With the liberal use of chronic, indwelling ureteral stents, extensive pelvic surgery, radiotherapy, and vascular pathology an increase of the incidence has been noted. Diagnosis remains a challenge because conventional radiographic tests are often unsuccessful in identifying the fistula. Hematuria might not be associated directly with an UAF, however it should be on the differential diagnosis list in patients with predisposing risk factors. Endoluminal repair using a stent graft represents a minimally invasive treatment that can be performed safely even in a hostile abdomen. A longer followup will be necessary for definitive long-term results.
